# Acyl‐CoA‐binding proteins: bridging long‐chain acyl‐CoA metabolism to gene regulation

**DOI:** 10.1111/nph.70142

**Published:** 2025-04-22

**Authors:** Allegra Wundersitz, Kurt M. V. Hoffmann, Joost T. van Dongen

**Affiliations:** ^1^ Department of Biology, Molecular Ecology of the Rhizosphere RWTH Aachen University 52074 Aachen Germany; ^2^ Department of Biology RWTH Aachen University 52074 Aachen Germany

**Keywords:** acyl‐CoA‐binding proteins, long‐chain acyl‐CoA signaling, metabolic regulation, plant hypoxia signaling, stress responses, unsaturated lipids

## Abstract

Acyl‐Coenzyme A‐binding proteins (ACBPs) sequester and transport long‐chain acyl‐Coenzyme A (LCA‐CoA) molecules, key intermediates in lipid metabolism, membrane biogenesis, and energy production. In addition, recent research emphasizes their regulatory role in linking the metabolic state to gene expression. In animals, ACBPs coordinate acetyl‐CoA metabolism and enzyme activity, thereby affecting gene expression through broad signaling networks. In plants, ACBPs contribute to development and stress responses, with hypoxia research showing their involvement in detecting LCA‐CoA fluctuations to trigger genetic acclimation. This review explores ACBPs in LCA‐CoA signaling and gene regulation, emphasizing their function as universal ‘translators’ of metabolic states for cellular acclimation. Further ACBP research will offer novel regulatory insights into numerous signaling pathways fundamental to health, development, and environmental responses across kingdoms.

**Table:** 

	Contents	
	Summary	1960
I.	Introduction	1960
II.	ACBP and LCA‐CoA research in animals	1963
III.	ACBPs in plant stress responses	1964
IV.	Sensing hypoxia: ACBPs as metabolic translators	1964
V.	Conclusions and future perspectives	1965
	Acknowledgements	1965
	References	1965

## I. Introduction

Long‐chain acyl‐Coenzyme A (LCA‐CoA) esters are versatile lipid intermediates in processes, such as energy metabolism, complex lipid synthesis, and membrane remodeling. Beyond these roles, LCA‐CoAs emerged as signaling molecules that regulate ion channels, enzymes, gene expression, and stress responses (Neess *et al*., 2015; Islinger *et al*., 2020). These molecules are synthesized by LCA‐CoA synthetase (LACS in plants; ACSL in mammals), which activate long‐chain fatty acids (C_14_–C_22_) via ATP‐dependent thioesterification with CoA (Grevengoed *et al*., 2014). Owing to their amphipathic nature, LCA‐CoAs require transport proteins to avoid interfering with membrane and protein integrity. Evolutionary conserved acyl‐CoA‐binding proteins (ACBPs) play a central role in this context by binding LCA‐CoAs with high affinity (Box 1), preventing their harmful buildup while delivering them to specific metabolic processes (Grevengoed *et al*., 2014; Neess *et al*., 2015; Islinger *et al*., 2020).

Box 1 **Conserved ACB domains: sensors for LCA‐CoA dynamics?**

Acyl‐Coenzyme A‐binding (ACB) domains are roughly 10‐kDa‐sized phylogenetically conserved protein domains that are found across all eukaryotic kingdoms (plants, fungi, and animals), as well as some eubacteria (Neess *et al*., 2015; Islinger *et al*., 2020). ACB domains are characterized by a four *α*‐helix bundle that folds into a shell‐like framework and exhibits high affinity for a wide range of long‐chain (C_14_‐C_22_) acyl‐Coenzyme A (LCA‐CoA) molecules (Neess *et al*., 2015; Islinger *et al*., 2020). Binding of LCA‐CoA is primarily mediated by polar residues on the surface of ACB domains that interact with the CoA portion, while a lipophilic region within the domain accommodates the acyl‐chain. Broadly, three bondings are relevant for LCA‐CoA binding to ACB domains: (1) binding to the 3′‐ribophosphate; (2) interactions with the adenosine moiety; and (3) hydrophobic interactions with the acyl‐chain (Islinger *et al*., 2020). This arrangement sequesters LCA‐CoA molecules, protecting them from hydrolysis and nonspecific interactions with their cellular environment. Subtle variations in amino acid composition among ACB isoforms result in shifts in binding preferences toward LCA‐CoAs. This capability positions ACB domains as potential sensors of minor metabolic fluctuations in LCA‐CoA pool compositions. Notably, structural data (available via the Protein Data Bank) reveal that ligand binding to ACB domains does not trigger conformational changes. This suggests that ACBP‐mediated LCA‐CoA signaling likely relies on competitive binding or steric effects introduced by specific LCA‐CoA species, which influence interactions between ACBP and other proteins. Future research should focus on uncovering the structural and functional details of ACB‐LCA‐CoA interactions and identify additional interaction partners of these complexes. Such studies will help to fully elucidate their signaling roles and to determine the broader scope and impact of these complexes in cellular regulatory networks.

**Fig. 1:**
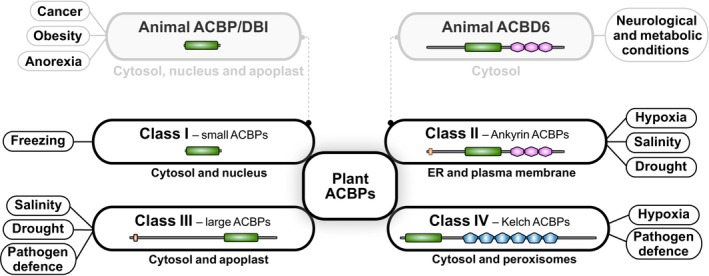
Functional and structural diversification of acyl‐Coenzyme A binding proteins (ACBPs) discussed in this review. ACBPs are a conserved protein family across biological kingdoms. In plants, ACBPs are grouped into four classes, each with unique subcellular localizations and roles in responding to diverse biotic and abiotic stresses. In animals, structural orthologs of Class I and Class II ACBPs carry out essential cellular functions, with their dysfunction linked to cancer, metabolic disorders, and neurological diseases. Green, conserved acyl‐CoA‐binding domain; orange, transmembrane/signal motif; pink, Ankyrin repeats; blue, Kelch repeats; DBI, diazepam‐binding inhibitor; ER, endoplasmic reticulum.

**Fig. 2:**
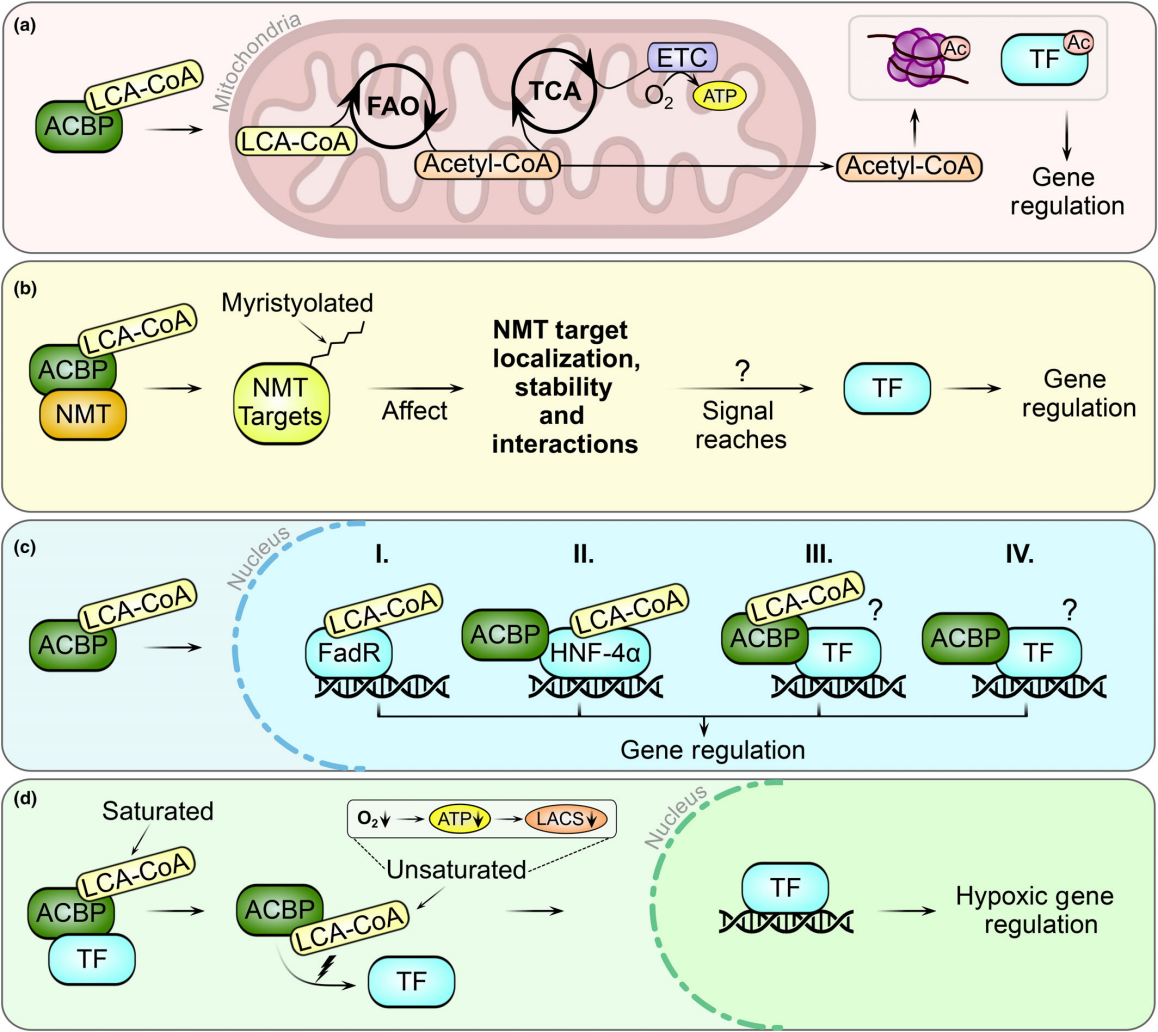
Acyl‐Coenzyme A‐binding protein (ACBP)–long‐chain acyl‐Coenzyme A (LCA‐CoA) complexes in gene regulatory processes. Four distinct modes of action have been identified through which ACBPs in association with LCA‐CoA molecules modulate gene expression across species. (a) ACBPs transport LCA‐CoAs to mitochondria, where they are used in fatty acid oxidation (FAO) to serve as acetyl‐CoA substrates in the tricarboxylic acid (TCA) cycle. This process drives the electron transport chain (ETC) to produce ATP via oxidative phosphorylation. Alternatively, FAO‐derived acetyl‐CoA can be transported into the cytosol and nucleus, serving as a substrate for histone and transcription factor (TF) acetylation (Ac), thereby regulating gene expression. (b) ACBPs interact with N‐myristoyltransferases (NMTs), protecting these enzymes from interference by more abundant nonmyristoyl‐CoAs and enhancing their activity. NMT‐mediated myristoylation modifies the localization, stability, and interactions of target proteins, influencing regulatory signaling pathways that may ultimately converge on transcriptional regulation via currently unknown mechanisms. (c) ACBPs transport LCA‐CoAs into the nucleus, where these molecules bind TFs, such as bacterial fatty acid metabolism regulator (FadR) (I) or mammalian hepatocyte nuclear factor‐4alpha (HNF‐4α) (II), to modulate their DNA‐binding activity. Additionally, ACBPs may interact directly with TFs, either in association with LCA‐CoAs (III), or the protein complex is fully functional even without interaction with LCA‐CoA as cofactor (IV). However, the dynamics and molecular consequences of these interactions remain poorly understood. (d) Binding of saturated and unsaturated LCA‐CoA species to ACBPs affects their interaction with TFs under varying physiological conditions. During hypoxia, reduced ATP levels decrease LCA‐CoA synthetase (LACS) activity, altering the LCA‐CoA pool to favor unsaturated LCA‐CoA species. This shift leads to the dissociation of TFs from ACBPs, enabling their nuclear translocation and activation of hypoxic gene expression.

The ACBP family comprises single‐ and multi‐domain proteins, with different domain‐mediating roles, such as protein–protein interactions and directing subcellular localization (Fig. 1). ACBPs not only participate in LCA‐CoA trafficking, but also engage in extracellular signaling, organelle tethering, enzyme modulation, and gene regulation across kingdoms (Fig. 2) (Islinger *et al*., 2020). Owing to these broad involvements, malfunctioning of ACBPs results in various human diseases, including cancer, neurodevelopmental, and metabolic disorders, whereas in plants, their dysfunction is associated with impaired development and altered stress tolerance (Duman *et al*., 2019; Hu *et al*., 2023; Montegut *et al*., 2023; Du *et al*., 2024; Kaiyrzhanov *et al*., 2024). However, while their importance in LCA‐CoA transport and metabolism is largely recognized, ACBP functionality in response to LCA‐CoA fluctuations often remains unexplored.

LCA‐CoA pools, composed of diverse species varying in chain length and degree of unsaturation, are dynamically regulated across subcellular compartments and tissues (Box 2). Distinct LACS/ACSL isoforms play a central role in forming these pools by generating LCA‐CoAs with individual fates tailored to specific subcellular localizations (Grevengoed *et al*., 2014; Wang *et al*., 2021, 2024). Furthermore, ACBP isoforms shape these pools by selectively sequestering specific species (Box 1), thereby passively fine‐tuning LCA‐CoA availability for cellular processes. These dynamics are highly responsive to physiological and pathophysiological states, including diabetes and hypoxia, the latter of which shifts the ratio from saturated to unsaturated LCA‐CoAs (Schmidt *et al*., 2018; Shao *et al*., 2024; Striesow *et al*., 2024; Wang *et al*., 2024).

Box 2 **Shedding light on the invisible: recent insights into LCA‐CoA dynamics**

Long‐chain acyl‐Coenzyme A (LCA‐CoA) pools respond rapidly to metabolic and environmental cues, such as nutrient availability, stress, and cellular energy status. However, their low abundance, instability, rapid turnover, and complex subcellular compartmentalization present immense challenges for accurate measurements.

Recent advances in compartment‐specific metabolomics and biosensors have enabled detailed investigations of subcellular LCA‐CoA dynamics (Trefely *et al*., 2022; Wang *et al*., 2024). For example, stable isotope labeling of essential nutrients in cell culture–subcellular fractionation (SILEC‐SF) improves acyl‐CoA quantification by embedding isotope‐labeled standards throughout the fractionation process, minimizing sample loss and processing inconsistencies (Trefely *et al*., 2022). SILEC‐SF demonstrated compartment‐specific metabolic changes, such as reduced mitochondrial succinyl‐CoA levels during hypoxia. However, its reliance on cell‐line standards limits applicability in complex tissues with metabolic heterogeneity (Trefely *et al*., 2022).

Alternatively, the high response of long‐chain acyl‐CoA (LACSerHR) biosensor by Wang *et al*. (2021, 2024) enables real‐time, *in vivo* monitoring of LCA‐CoA fluctuations, revealing enzyme‐ or stress‐induced shifts in LCA‐CoA pools. For instance, the biosensor confirmed LCA‐CoA synthetase 4 (ACSL4)‐dependent LCA‐CoA changes during ferroptosis and fluctuations in LCA‐CoA metabolism in type 2 diabetes (Wang *et al*., 2024). Based on the bacterial fatty acid metabolism regulator transcription factor, which binds LCA‐CoAs to modulate its ability to bind DNA (Fig. 2c), LACSerHR uses a conformation‐sensitive yellow fluorescent protein (cpYFP) to measure fluorescence changes upon LCA‐CoA binding (van Aalten *et al*., 2001; Petrescu *et al*., 2003; Wang *et al*., 2024). While powerful, LACSerHR cannot distinguish individual LCA‐CoA species and is affected by pH sensitivity, which impacts measurement accuracy in some settings.

Both SILEC‐SF and LACSerHR have shown that LCA‐CoA pools undergo subcellular changes during hypoxia, ferrotopsis, and type 2 diabetes. However, challenges remain in making these techniques applicable to complex tissues and distinguishing between individual species. Future tools should aim for species‐specific detection and enhanced accuracy to further unravel the signaling roles of LCA‐CoAs.

Plant hypoxia research revealed that ACBPs sense shifts in the cytosolic LCA‐CoA pool and directly transmit this information to interacting transcription factors, enabling their release to initiate hypoxic gene expression (Fig. 2d) (Schmidt *et al*., 2018; Zhou *et al*., 2020; Guo *et al*., 2024). This mechanism illustrates that the interaction between ACBPs and LCA‐CoAs can provide a nuanced communication channel between the metabolic state of the cell and the gene regulatory network. In animals, ACBP research highlights both LCA‐CoA‐independent and LCA‐CoA‐dependent signaling roles, with the latter influencing gene expression through diverse signaling pathways (Fig. 2a–c) (Joseph *et al*., 2020; Soupene *et al*., 2020; Duman *et al*., 2023; Li & Karpac, 2023).

This review summarizes recent advances in ACBP research across kingdoms, focusing on their roles in linking LCA‐CoA metabolism to gene regulation (Fig. 2). By including nonplant research, we seek to provide a comprehensive perspective on ACBP functionality, offering insights into conserved roles while introducing developing technologies that could transform ACBP research in plants (Box 2).

## II. ACBP and LCA‐CoA research in animals

Among the ACBP isoforms in animals, mammalian ACBP/diazepam‐binding inhibitor (DBI) consists solely of the conserved acyl‐CoA‐binding (ACB) domain and functions both intra‐ and extracellularly (Box 1; Fig. 1: Animal ACBP/DBI) (Montegut *et al*., 2023). Intracellularly, ACBP/DBI is involved in linking the metabolic state of the cell with gene regulation via modulating acetyl‐CoA pools (Fig. 2a). ACBP/DBI is highly expressed in many cancers, where it mediates the transport of LCA‐CoAs to mitochondria, fueling fatty acid oxidation to support tumor growth and invasiveness (Duman *et al*., 2019). Recently, Duman *et al*. (2023) reported that ACBP/DBI knockdown reduces invasion and proliferation of glioblastoma, the most common malignant brain tumor in adults. This reduction is accompanied by widespread changes in gene expression, suggesting that ACBP/DBI plays a role not only in energy production, but also in driving transcriptional reprogramming (Duman *et al*., 2023). Their study proposed that the transcriptional changes following ACBP/DBI knockdown result from reduced acetyl‐CoA production, impairing histone acetylation and thereby altering gene expression (Duman *et al*., 2023). Supporting this concept, Li & Karpac (2023) found that *Dm*Acbp6, the structural analog of ACBP/DBI in fruit fly (*Drosophila melanogaster*), is involved in regulating acetyl‐CoA levels to initiate transcriptional reprogramming for nutrient‐dependent midgut resizing. The *Dm*Acbp6‐mediated modulation of the acetyl‐CoA pool affects acetylation of various proteins, such as the *Dm*STAT92e transcription factor, promoting intestinal stem cell proliferation to support nutrient‐responsive tissue plasticity (Li & Karpac, 2023). These findings position ACBPs as regulators of acetyl‐CoA metabolism that indirectly leads to modulation of gene expression across species.

Beyond their role as indirect modulators, ACBPs also directly shape metabolism via regulating enzyme activities through protein–protein interactions. ACBD6, a multi‐domain ACBP with a C‐terminal ankyrin‐repeat (ANK) domain (Fig. 1: Animal ACBD6), regulates protein N‐myristoylation, a co‐ and posttranslational modification that influences target stability, localization, and function (Soupene *et al*., 2016, 2020; Soupene & Kuypers, 2022). Dysfunction of ACBD6 has been associated with neurological and metabolic conditions linked to perturbed N‐myristoylated protein networks, highlighting a critical role for ACBD6 in maintaining proper organismal health (Yeetong *et al*., 2023; Kaiyrzhanov *et al*., 2024). Through its ANK domain, ACBD6 interacts with N‐myristoyltransferases (NMTs) and shields these enzymes from inhibition by more abundant nonmyristoyl LCA‐CoAs, thereby enhancing NMT activity (Soupene *et al*., 2020). This protective interaction with NMTs allows ACBD6 to affect multiple signaling pathways that could converge on transcription factor activity (Fig. 2b). However, while proteomic studies in ACBD6‐deficient zebrafish (*Danio rerio*) and frog (*Xenopus tropicalis*) cells suggest broad impacts on RNA processing and stress responses (Kaiyrzhanov *et al*., 2024), direct evidence linking ACBD6 to transcriptional regulation remains limited.

Notably, while roles in regulating acetyl‐CoA levels and NMT activity are tied to LCA‐CoA binding (Fig. 2a,b), not all of their functions depend on this interaction. For instance, extracellular ACBP/DBI acts as a neuropeptide that binds γ‐aminobutyric acid type A receptors, affecting processes, such as neurogenesis, apoptosis, and appetite stimulation (Alquier *et al*., 2021; Montegut *et al*., 2023). Aligning with its role in appetite stimulation, extracellular ACBP/DBI levels are elevated in obese and reduced in anorexic patients, positioning it as a promising therapeutic target for anorexia (Fig. 1) (Bravo‐San Pedro *et al*., 2019; Charmpilas *et al*., 2020; Chen *et al*., 2024). In this context, research showed that mutations reducing the affinity of ACBP/DBI for LCA‐CoAs do not impair its appetite‐stimulatory effect, suggesting that at least some functions of ACBP/DBI operate independent of LCA‐CoA binding (Joseph *et al*., 2020).

In summary, ACBPs in nonplant species play diverse roles in regulating acetyl‐CoA pools, transcriptional reprogramming, enzyme activity, and extracellular signaling. However, key questions remain regarding their contributions to transcriptional regulation and how these processes intersect with binding of specific LCA‐CoAs. Research in plants, where ACBPs regulate development and stress responses, has revealed species‐specific effects of distinct LCA‐CoAs on their regulatory functions, providing novel insights that may bridge gaps in our understanding of ACBP functionality across kingdoms.

## III. ACBPs in plant stress responses

Based on phylogeny and protein domains, plant ACBPs fall into four classes: small ACBPs (Class I), ANK‐ACBPs (Class II), large ACBPs (Class III), and Kelch‐ACBPs (Class IV) (Fig. 1: Plant ACBPs) (Meng *et al*., 2011). Class I comprises cytoplasmic and nuclear ACBPs that contain only an ACB domain. Although structurally resembling mammalian ACBP/DBI, functional data on plant orthologs remain limited to conferring freezing tolerance upon overexpression (Chen *et al*., 2008). Class II ACBPs feature, in addition to the ACB domain, C‐terminal ANK repeats and an N‐terminal transmembrane motif that targets them to the endoplasmic reticulum (ER) and plasma membrane. Class III‐members are high molecular weight ACBPs with a C‐terminal ACB domain and target to the apoplast, whereas Class IV ACBPs have C‐terminal Kelch motifs that engage in protein–protein interactions and localize to the cytosol or peroxisomes (Meng *et al*., 2014).

Recently, these classes have been characterized in barley (Chang *et al*., 2024), wheat (Hu *et al*., 2023), cotton (Chen *et al*., 2023), poplar (Chang *et al*., 2022), and many legumes (Ling *et al*., 2023; Du *et al*., 2024), with transcript analyses highlighting tissue‐specific expression and responsiveness to both environmental and developmental cues. For instance, in barley (*Hordeum vulgare*), *HvACBPs* are differentially induced by phytohormones and abiotic stresses, suggesting roles in stress acclimation and development (Chang *et al*., 2024). However, although ACBPs participate in various regulatory pathways, the precise molecular functions across different classes remain ambiguous and seem to partly overlap.

Class III and IV ACBPs were implicated to be involved in autophagy‐related processes linked to pathogen defense, though their molecular role in this context remains unclear (Fig. 1). In wheat (*Triticum aestivum*), *TaACBP4A‐1* (Class III) and *TaACBP4A‐2* (Class IV) are induced by fungal infection with *Blumeria graminis* f. sp. *tritici* (*Bgt*) (Hu *et al*., 2023). Silencing of *TaACBP4A‐1* and *TaACBP4A‐2* increases susceptibility to *Bgt*, suggesting that both Class III and Class IV *Ta*ACBPs contribute to pathogen defense. Yeast two‐hybrid assays indicate that *Ta*ACBP4A‐1 interacts with the autophagy‐related protein *Ta*ATG8g, implying a role for *Ta*ACBP4A‐1 in autophagy (Hu *et al*., 2023). In previous studies using the model plant *Arabidopsis thaliana*, overexpression of Class III *At*ACBP3 led to destabilization of *At*ATG8, with mutant plants displaying accelerated leaf senescence alongside impaired autophagy (Xiao *et al*., 2010). These findings indicate a conserved role for Class III ACBPs in regulating autophagy across plants through a process involving ATG8 and its destabilization. However, additional evidence verifying the *Ta*ACBP4A‐1–*Ta*ATG8g interaction is currently missing, and the functional role of this complex remains to be determined.

Class II and III ACBPs have been implicated in responses to abiotic stresses, such as drought, salinity, and hypoxia, but many aspects of their molecular mechanisms remain unresolved (Fig. 1). For instance, in *Medicago truncatula* and *Medicago sativa*, *Mt/MsACBP1* (Class III) and *Mt/MsACBP2* (Class II) are induced by high salinity, with *Mt/MsACBP2* overexpression enhancing salt and drought tolerance in *Arabidopsis* (Du *et al*., 2024). In soybean (*Glycine max*), a proposed mechanism driving the ACBP‐mediated salt stress resilience involves Class II *Gm*ACBPs that interact with lipoxygenase (*Gm*LOX) via linolenoyl‐CoA (C18:3‐CoA) (Lung *et al*., 2022). This interaction forms a complex that sequesters *Gm*LOX at the ER and plasma membrane under nonstressed conditions (Lung *et al*., 2022). Under salinity, increased phosphatidic acid (PA) contents compete with linolenoyl‐CoA for binding to *Gm*ACBPs, leading to *Gm*LOX release and activation of oxylipin signaling, which is essential for stress resistance (Lung *et al*., 2022). However, it is currently unclear whether and how PA binds to the ACB domain to compete with the higher‐affinity linolenoyl‐CoA ligand. Based on *in vitro* kinase assays and computational predictions, further regulatory layers may involve phosphorylation of Class II *Gm*ACBPs, but *in vivo* evidence and functional consequences of this modification remain unexplored (Moradi *et al*., 2024).

Overall, ACBPs are regulators of various processes, including development, autophagy, and stress responses. While their ability to interact with different lipid and LCA‐CoA species positions them as central hubs in lipid signaling (Box 1), many molecular consequences of their interplay remain unclear. Strikingly, hypoxia research has demonstrated that specific LCA‐CoA species act as molecular triggers that induce differential gene expression clusters in *Arabidopsis* (Schmidt *et al*., 2018). By integrating these metabolic signals, ACBPs serve as pivotal connectors that directly bridge lipid metabolism to gene regulation for cellular acclimation.

## IV. Sensing hypoxia: ACBPs as metabolic translators

ACBPs are involved in initiating the onset of hypoxic gene expression by sensing and translating cytoplasmic LCA‐CoA shifts into transcriptional responses (Schmidt *et al*., 2018; Zhou *et al*., 2020; Guo *et al*., 2024). Hypoxia hampers oxygen‐dependent processes and triggers a plethora of molecular responses to restore cellular homeostasis. One aspect of these metabolic shifts involves the accumulation of unsaturated LCA‐CoA species, which act as signaling molecules through interactions with ACBPs to initiate hypoxic gene expression (Fig. 2d) (Schmidt *et al*., 2018; Zhou *et al*., 2020; Guo *et al*., 2024; Shao *et al*., 2024; Striesow *et al*., 2024).

Upon hypoxia, the relative abundance of oleoyl‐CoA (C18:1‐CoA) increases because LACS activity is reduced under low ATP conditions. This shift in the composition of the LCA‐CoA pool thus creates an energy‐dependent signal that leads to the dissociation of the subgroup VII ethylene‐response transcription factor (ERFVII) related to apetala 2.12 (RAP2.12) from Class II ACBPs (Schmidt *et al*., 2018). The release allows RAP2.12 to translocate into the nucleus and activate genes required for anaerobic metabolism (Licausi *et al*., 2011; Schmidt *et al*., 2018). In addition to reduced LACS activity, increased *fatty acid desaturase* expression during hypoxia increases the level of polyunsaturated linolenoyl‐CoA (C18:3‐CoA), which similarly triggers the release of RAP2.12 from Class II ACBPs (Klinkenberg *et al*., 2014; Zhou *et al*., 2020; Shao *et al*., 2024). These observations suggest that various unsaturated LCA‐CoAs contribute to fine‐tuning metabolic responses under hypoxic stress, possibly conveying nuanced metabolic states. Recently, Guo *et al*. (2024) identified a related mechanism where oleoyl‐CoA releases the WRKY70 transcription factor from ACBP4 (Class IV), leading to increased *RAP2.12* expression and enhancement of the hypoxic response. Together, these findings suggest a broad role for different classes of ACBPs in linking LCA‐CoA signaling to transcription factor activity during metabolic acclimation to hypoxia (Fig. 2d).

## V. Conclusions and future perspectives

ACBPs are versatile cellular regulators across kingdoms, transitioning from simple LCA‐CoA transporters to extracellular signal transducers, enzymatic modulators, and mediators of LCA‐CoA‐dependent transcriptional reprogramming (Figs 1, 2). Their ability to bind, transport, and translate LCA‐CoA signals contributes to a broad range of cellular functions, including nutrient‐responsive regulation and the modulation of transcription factor activity. However, roles such as the appetite‐stimulatory effects of extracellular ACBP/DBI, appear independent of LCA‐CoA binding, underscoring their functional versatility.

The observed versatility of ACBPs in gene regulation is particularly intriguing, involving mechanisms, such as modulating acetyl‐CoA availability, enzyme regulation, and transcription factor interactions (Fig. 2). In plants, their role during hypoxia signaling demonstrates their capacity to sense and communicate cellular metabolic states to transcription factors, allowing precise gene expression adjustments. Beyond hypoxia, ACBPs participate in other abiotic stresses like drought and salinity, where shifts in lipid metabolism create unique LCA‐CoA signatures (He & Ding, 2020). Thus, it is tempting to speculate whether ACBPs may act as general metabolic sensors that translate LCA‐CoA signatures generated by the cell to their protein interaction partners.

The versatility of ACBPs in binding diverse LCA‐CoAs and interacting with proteins across various cellular contexts suggests their regulatory roles may extend beyond our current understanding. However, the precise mechanism underlying LCA‐CoA‐mediated complex destabilization and the spectrum of their protein interactions remain unclear. Techniques, such as protein pull‐downs, mass spectrometry, compartment‐specific metabolomics, and LCA‐CoA biosensors, offer potential to uncover these dynamics (Box 2). These approaches could clarify whether ACBPs act as general coordinators of cellular acclimation or specialize in specific processes, such as oxygen sensing in plants. Addressing these questions may redefine ACBPs as universal metabolic translators, opening exciting avenues for understanding their roles across kingdoms.

## Competing interests

None declared.

## Disclaimer

The New Phytologist Foundation remains neutral with regard to jurisdictional claims in maps and in any institutional affiliations.

## References

[nph70142-bib-0001] van Aalten DM , DiRusso CC , Knudsen J . 2001. The structural basis of acyl coenzyme A‐dependent regulation of the transcription factor FadR. European Molecular Biology Organization 20: 2041–2050. 10.1093/emboj/20.8.2041 PMC125426 11296236

[nph70142-bib-0002] Alquier T , Christian‐Hinman CA , Alfonso J , Faergeman NJ . 2021. From benzodiazepines to fatty acids and beyond: revisiting the role of ACBP/DBI. Trends in Endocrinology and Metabolism 32: 890–903. 34565656 10.1016/j.tem.2021.08.009 PMC8785413

[nph70142-bib-0003] Bravo‐San Pedro JM , Sica V , Martins I , Pol J , Loos F , Maiuri MC , Durand S , Bossut N , Aprahamian F , Anagnostopoulos G *et al*. 2019. Acyl‐CoA‐binding protein is a lipogenic factor that triggers food intake and obesity. Cell Metabolism 30: 754–767. 31422903 10.1016/j.cmet.2019.07.010

[nph70142-bib-0004] Chang H , Ma M , Gu M , Li S , Li M , Guo G , Xing G . 2024. *Acyl‐CoA‐binding protein* (*ACBP*) genes involvement in response to abiotic stress and exogenous hormone application in barley (*Hordeum vulgare* L.). BMC Plant Biology 24: 236–249. 38561660 10.1186/s12870-024-04944-6 PMC10985865

[nph70142-bib-0005] Chang Y , Xu X , Zheng H , Xie H , Li B , Chen S , Li Y , Dai S . 2022. Genome‐wide identification and characterization of *ACBP* gene family in Populus reveal salinity alkali‐responsive profiles. Journal of Forestry Research 34: 481–496.

[nph70142-bib-0006] Charmpilas N , Ruckenstuhl C , Sica V , Buttner S , Habernig L , Dichtinger S , Madeo F , Tavernarakis N , Bravo‐San Pedro JM , Kroemer G . 2020. Acyl‐CoA‐binding protein (ACBP): a phylogenetically conserved appetite stimulator. Cell Death & Disease 11: 7. 31907349 10.1038/s41419-019-2205-x PMC6944704

[nph70142-bib-0007] Chen H , Moriceau S , Joseph A , Mailliet F , Li S , Tolle V , Duriez P , Dardennes R , Durand S , Carbonnier V *et al*. 2024. Acyl‐CoA binding protein for the experimental treatment of anorexia. Science Translational Medicine 16: 715. 10.1126/scitranslmed.adl0715 39141698

[nph70142-bib-0008] Chen QF , Xiao S , Chye ML . 2008. Overexpression of the *Arabidopsis* 10‐kilodalton *acyl‐coenzyme A‐binding protein ACBP6* enhances freezing tolerance. Plant Physiology 148: 304–315. 18621979 10.1104/pp.108.123331 PMC2528132

[nph70142-bib-0009] Chen Y. Fu M. Li H. Wang L. Liu R. Liu Z. 2023 Molecular characterization of the *Acyl‐CoA‐Binding Protein* genes reveals their significant roles in oil accumulation and abiotic stress response in cotton Genes 14: 859 37107617 10.3390/genes14040859 PMC10137972

[nph70142-bib-0010] Du W , Huang H , Kong W , Jiang W , Pang Y . 2024. Over‐expression of *Medicago Acyl‐CoA‐binding 2* genes enhance salt and drought tolerance in *Arabidopsis* . International Journal of Biological Macromolecules 268: 131631. 38631584 10.1016/j.ijbiomac.2024.131631

[nph70142-bib-0011] Duman C , Di Marco B , Nevedomskaya E , Ulug B , Lesche R , Christian S , Alfonso J . 2023. Targeting fatty acid oxidation via Acyl‐CoA binding protein hinders glioblastoma invasion. Cell Death and Disease 14: 296. 37120445 10.1038/s41419-023-05813-0 PMC10148872

[nph70142-bib-0012] Duman C , Yaqubi K , Hoffmann A , Acikgoz AA , Korshunov A , Bendszus M , Herold‐Mende C , Liu HK , Alfonso J . 2019. Acyl‐CoA‐binding protein drives glioblastoma tumorigenesis by sustaining fatty acid oxidation. Cell Metabolism 30: 274–289. 31056285 10.1016/j.cmet.2019.04.004

[nph70142-bib-0013] Grevengoed TJ , Klett EL , Coleman RA . 2014. Acyl‐CoA metabolism and partitioning. Annual Review of Nutrition 34: 1–30. 10.1146/annurev-nutr-071813-105541 PMC5881898 24819326

[nph70142-bib-0014] Guo M , Yao Y , Yin K , Tan L , Liu M , Hou J , Zhang H , Liang R , Zhang X , Yang H *et al*. 2024. ACBP4‐WRKY70‐RAP2.12 module positively regulates submergence‐induced hypoxia response in *Arabidopsis thaliana* . Journal of Integrative Plant Biology 66: 1052–1067. 38501444 10.1111/jipb.13647

[nph70142-bib-0015] He M , Ding N‐Z . 2020. Plant unsaturated fatty acids: multiple roles in stress response. Frontiers in Plant Science 11: 562785. 33013981 10.3389/fpls.2020.562785 PMC7500430

[nph70142-bib-0016] Hu P , Ren Y , Xu J , Luo W , Wang M , Song P , Guan Y , Hu H , Li C . 2023. Identification of *acyl‐CoA‐binding protein* gene in *Triticeae* species reveals that *Ta*ACBP4A‐1 and *Ta*ACBP4A‐2 positively regulate powdery mildew resistance in wheat. International Journal of Biological Macromolecules 246: 125526. 37379955 10.1016/j.ijbiomac.2023.125526

[nph70142-bib-0017] Islinger M , Costello JL , Kors S , Soupene E , Levine TP , Kuypers FA , Schrader M . 2020. The diversity of ACBD proteins – from lipid binding to protein modulators and organelle tethers. BBA – Molecular Cell Research 1867: 118675. 32044385 10.1016/j.bbamcr.2020.118675 PMC7057175

[nph70142-bib-0018] Joseph A , Moriceau S , Sica V , Anagnostopoulos G , Pol J , Martins I , Lafarge A , Maiuri MC , Leboyer M , Loftus J *et al*. 2020. Metabolic and psychiatric effects of acyl coenzyme A binding protein (ACBP)/diazepam binding inhibitor (DBI). Cell Death & Disease 11: 502. 32632162 10.1038/s41419-020-2716-5 PMC7338362

[nph70142-bib-0019] Kaiyrzhanov R , Rad A , Lin SJ , Bertoli‐Avella A , Kallemeijn WW , Godwin A , Zaki MS , Huang K , Lau T , Petree C *et al*. 2024. Bi‐allelic *ACBD6* variants lead to a neurodevelopmental syndrome with progressive and complex movement disorders. Brain 147: 1436–1456. 37951597 10.1093/brain/awad380 PMC10994533

[nph70142-bib-0020] Klinkenberg J , Faist H , Saupe S , Lambertz S , Krischke M , Stingl N , Fekete A , Mueller MJ , Feussner I , Hedrich R *et al*. 2014. Two fatty acid desaturases, STEAROYL‐ACYL CARRIER PROTEIN Δ9‐DESATURASE6 and FATTY ACID DESATURASE3, are involved in drought and hypoxia stress signaling in *Arabidopsis* crown galls. Plant Physiology 164: 570–583. 24368335 10.1104/pp.113.230326 PMC3912090

[nph70142-bib-0021] Li X , Karpac J . 2023. A distinct Acyl‐CoA binding protein (ACBP6) shapes tissue plasticity during nutrient adaptation in *Drosophila* . Nature Communications 14: 7599. 10.1038/s41467-023-43362-4 PMC10663470 37989752

[nph70142-bib-0022] Licausi F , Kosmacz M , Weits DA , Giuntoli B , Giorgi FM , Voesenek LA , Perata P , van Dongen JT . 2011. Oxygen sensing in plants is mediated by an N‐end rule pathway for protein destabilization. Nature 479: 419–422. 22020282 10.1038/nature10536

[nph70142-bib-0023] Ling J , Li L , Lin L , Xie H , Zheng Y , Wan X . 2023. Genome‐wide identification of *acyl‐CoA binding proteins* and possible functional prediction in legumes. Frontiers in Genetics 13: 1057160. 36704331 10.3389/fgene.2022.1057160 PMC9871394

[nph70142-bib-0024] Lung SC , Lai SH , Wang H , Zhang X , Liu A , Guo ZH , Lam HM , Chye ML . 2022. Oxylipin signaling in salt‐stressed soybean is modulated by ligand‐dependent interaction of Class II acyl‐CoA‐binding proteins with lipoxygenase. Plant Cell 34: 1117–1143. 34919703 10.1093/plcell/koab306 PMC8894927

[nph70142-bib-0025] Meng W , Hsiao AS , Gao C , Jiang L , Chye ML . 2014. Subcellular localization of rice acyl‐CoA‐binding proteins (ACBPs) indicates that *Os*ACBP6::GFP is targeted to the peroxisomes. New Phytologist 203: 469–482. 24738983 10.1111/nph.12809

[nph70142-bib-0026] Meng W , Su YCF , Saunders RMK , Chye ML . 2011. The rice *acyl‐CoA‐binding protein* gene family: phylogeny, expression and functional analysis. New Phytologist 189: 1170–1184. 21128943 10.1111/j.1469-8137.2010.03546.x

[nph70142-bib-0027] Montegut L. Abdellatif M. Motino O. Madeo F. Martins I. Quesada V. Lopez‐Otin C. Kroemer G. 2023 Acyl coenzyme A binding protein (ACBP): an aging‐ and disease‐relevant “autophagy checkpoint” Aging Cell 22: e13910. 37357988 10.1111/acel.13910 PMC10497816

[nph70142-bib-0028] Moradi A , Lung SC , Chye ML . 2024. Interaction of Soybean (*Glycine max* (L.) Merr.) Class II ACBPs with MPK2 and SAPK2 Kinases: New Insights into the Regulatory Mechanisms of Plant ACBPs. Plants 13: 1146. 38674555 10.3390/plants13081146 PMC11055065

[nph70142-bib-0029] Neess D , Bek S , Engelsby H , Gallego SF , Faergeman NJ . 2015. Long‐chain acyl‐CoA esters in metabolism and signaling: Role of acyl‐CoA binding proteins. Progress in Lipid Research 59: 1–25. 25898985 10.1016/j.plipres.2015.04.001

[nph70142-bib-0030] Petrescu AD , Payne HR , Boedecker A , Chao H , Hertz R , Bar‐Tana J , Schroeder F , Kier AB . 2003. Physical and functional interaction of Acyl‐CoA‐binding protein with hepatocyte nuclear factor‐4 alpha. Journal of Biological Chemistry 278: 51813–51824. 14530276 10.1074/jbc.M303858200

[nph70142-bib-0031] Schmidt RR , Fulda M , Paul MV , Anders M , Plum F , Weits DA , Kosmacz M , Larson TR , Graham IA , Beemster GTS *et al*. 2018. Low‐oxygen response is triggered by an ATP‐dependent shift in oleoyl‐CoA in *Arabidopsis* . Proceedings of the National Academy of Sciences, USA 115: E12101. 10.1073/pnas.1809429115 PMC6304976 30509981

[nph70142-bib-0032] Shao D , Yu C , Chen Y , Qiu X , Chen J , Zhao H , Chen K , Wang X , Chen P , Gao G *et al*. 2024. Lipids signaling and unsaturation of fatty acids participate in ramie response to submergence stress and hypoxia‐responsive gene regulation. International Journal of Biological Macromolecules 263: 130104. 38350586 10.1016/j.ijbiomac.2024.130104

[nph70142-bib-0033] Soupene E , Kao J , Cheng DH , Wang D , Greninger AL , Knudsen GM , DeRisi JL , Kuypers FA . 2016. Association of NMT2 with the acyl‐CoA carrier ACBD6 protects the N‐myristoyltransferase reaction from palmitoyl‐CoA. Journal of Lipid Research 57: 288–298. 26621918 10.1194/jlr.M065003 PMC4727424

[nph70142-bib-0034] Soupene E , Kuypers FA . 2022. Dual role of ACBD6 in the acylation remodeling of lipids and proteins. Biomolecules 12: 1726. 36551154 10.3390/biom12121726 PMC9775454

[nph70142-bib-0035] Soupene E , Schatz UA , Rudnik‐Schoneborn S , Kuypers FA . 2020. Requirement of the acyl‐CoA carrier ACBD6 in myristoylation of proteins: activation by ligand binding and protein interaction. PLoS ONE 15: 229718. 10.1371/journal.pone.0229718 PMC7046191 32108178

[nph70142-bib-0036] Striesow J , Welle M , Busch LM , Bekeschus S , Wende K , Stohr C . 2024. Hypoxia increases triacylglycerol levels and unsaturation in tomato roots. BMC Plant Biology 24: 909. 39350052 10.1186/s12870-024-05578-4 PMC11441241

[nph70142-bib-0037] Trefely S , Huber K , Liu J , Noji M , Stransky S , Singh J , Doan MT , Lovell CD , von Krusenstiern E , Jiang H *et al*. 2022. Quantitative subcellular acyl‐CoA analysis reveals distinct nuclear metabolism and isoleucine‐dependent histone propionylation. Molecular Cell 82: 447–462. 34856123 10.1016/j.molcel.2021.11.006 PMC8950487

[nph70142-bib-0038] Wang W , Wang P , Zhu L , Liu B , Wei Q , Hou Y , Li X , Hu Y , Li W , Wang Y *et al*. 2024. An optimized fluorescent biosensor for monitoring long‐chain fatty acyl‐CoAs metabolism *in vivo* . Biosensors and Bioelectronics 247: 115935. 38128319 10.1016/j.bios.2023.115935

[nph70142-bib-0039] Wang W , Wei Q , Zhang J , Zhang M , Wang C , Qu R , Wang Y , Yang G , Wang J . 2021. A ratiometric fluorescent biosensor reveals dynamic regulation of long‐chain fatty acyl‐CoA esters metabolism. Angewandte Chemie International Edition 60: 13996–14004. 33837610 10.1002/anie.202101731

[nph70142-bib-0040] Xiao S , Gao W , Chen Q‐F , Chan S‐W , Zheng S‐X , Ma J , Wang M , Welti R , Chye M‐L . 2010. Overexpression of *Arabidopsis Acyl‐CoA Binding Protein ACBP3* promotes starvation‐induced and age‐dependent leaf senescence. Plant Cell 22: 1463–1482. 20442372 10.1105/tpc.110.075333 PMC2899868

[nph70142-bib-0041] Yeetong P , Tanpowpong N , Rakwongkhachon S , Suphapeetiporn K , Shotelersuk V . 2023. Neurodevelopmental disorder, obesity, pancytopenia, diabetes mellitus, cirrhosis, and renal failure in ACBD6‐associated syndrome: a case report. Neurology Genetics 9: e200046. 36457943 10.1212/NXG.0000000000200046 PMC9709716

[nph70142-bib-0042] Zhou Y , Tan WJ , Xie LJ , Qi H , Yang YC , Huang LP , Lai YX , Tan YF , Zhou DM , Yu LJ *et al*. 2020. Polyunsaturated linolenoyl‐CoA modulates ERF‐VII‐mediated hypoxia signaling in *Arabidopsis* . Journal of Integrative Plant Biology 62: 330–348. 31595698 10.1111/jipb.12875

